# Development of a Japanese Version of the Index of Sexual Satisfaction for Use in Couples with Young Children

**DOI:** 10.3390/bs12120503

**Published:** 2022-12-09

**Authors:** Ryoko Hidaka, Ikuko Sobue, Miki Yano, Ryoko Ito, Toshio Kobayashi

**Affiliations:** 1 Faculty of Health and Welfare, Prefectural University of Hiroshima, Hiroshima 723-0053, Japan; 2 Graduate School of Biomedical and Health Sciences, Hiroshima University, Hiroshima 734-8553, Japan; 3 Faculty of Health and Sciences, Hiroshima Cosmopolitan University, Hiroshima 734-0014, Japan; 4 School of Nursing and Health, Aichi Prefectural University, Aichi 480-1198, Japan

**Keywords:** sexual satisfaction, scale development, reliability and validity, transition to parenthood, couples with young children, Japan

## Abstract

The occurrence of sexual dysfunction in couples after childbirth is well recognized, yet sexual satisfaction in couples with young children (CYC) has received little research attention. This study sought to enable this construct to be measured by developing and validating a Japanese version of the Index of Sexual Satisfaction (ISS) in CYC. Data were collected using a self-administered questionnaire. Scale construction and validation were conducted using two independent samples drawn from 316 mothers and 272 fathers in Japan who had at least one child aged 6 or younger. Two underlying factors were identified using exploratory factor analysis: sexual satisfaction, measured by eight items, and sexual dissatisfaction, measured by three. Polychoric ordinal alpha coefficients indicated the reliability of the resulting scale (overall: 0.89, factor 1: 0.89, factor 2: 0.78), and confirmatory factor analysis and testing supported its validity, showing good model fit (goodness of fit index: 0.984, root mean square residual: 0.062) and satisfactory composite reliability (scale: 0.93, factor 1: 0.90, factor 2: 0.81) and average variance extracted (all ≥0.5). The Japanese version of the ISS for Couples with Young Children will be useful for investigating sexual satisfaction, which is essential to marital stability.

## 1. Introduction

A sexually satisfying life contributes not only to individual well-being [1,2] but also better relationship quality [3,4] and longevity [5]. Sexual satisfaction is therefore, an essential factor for promoting a stable married life [6,7,8].

However, the transition to parenthood can lead to a decrease in sexual well-being and, hence sexual satisfaction [9,10]. Pregnancy and childbirth can impair maternal sexual function (causing, e.g., perineal pain and decreased sexual desire and arousal) [11,12], impacting the couple’s sexual well-being. Fathers may also experience a decline in sexual function due to sexual interdependence with mothers [13,14]. Couples may engage in fewer sexual activities and do so less frequently, and both mothers and fathers may develop sexual concerns such as a mismatch of sexual desire and a change in body image [14]. A 2017 survey showed that 10.0% of couples in Japan become sexless after childbirth [15]. Thus, the sexual issues and satisfaction of couples in the postpartum period have been well studied and their prevalence well established.

Maternal difficulties with sexual function gradually resolve by 12 months postpartum [16,17], which may ease the sexual distress experienced by both parents. Nevertheless, decreased sexual function may continue for a longer period in some mothers. For example, a study finding that 77.3% of Japanese mothers reported sexual dysfunction 12 months after birth indicates that sexual adjustment takes 1–2 years [13]. Another study in Japan found that couples with at least one child aged under 3 reported a more sexless relationship than couples without children [18]. Further, factors such as sexual desire, frequency, and contentment have been shown to remain compromised up to 8 years after childbirth in some couples [19]. This suggests that sexual dissatisfaction extends well beyond the postpartum period for many couples with young children (CYC), which poses a threat to their sexual well-being and relational stability. These findings indicate a need for studies into whether CYC are sexually satisfied and which variables affect their sexual satisfaction. To the best of our knowledge, however, little research has been conducted on the sexual satisfaction of CYC. Moreover, any such research requires a valid measure of sexual satisfaction in CYC to be robust.

One of several measures used thus far to assess sexual satisfaction is the Index of Sexual Satisfaction (ISS) developed by Hudson et al. [20]. It is a concrete, easy-to-answer scale on sexual life and evaluates positive and negative aspects in detail. It has been used widely in research to evaluate couples’ global sexual satisfaction [21,22]. It could be considered a good fit to less sexually expressive cultures such as that of Japan. Japanese people can have difficulty disclosing sexual information [23,24]. Therefore, this measure is appropriate to use in investigating sexual satisfaction, as, in particular, the Japanese commonly and openly use most of the phrases in the ISS in their circles.

The original study that developed the index indicated that it had good internal consistency and discriminant validity in multi-ethnic participants in three populations in Hawaii who were aged 40–80, had a mean age of 25, and had a mean age of 37 [20]. Further studies showed it to have satisfactory reliability in newlywed couples in the USA [4,25] and long-term committed couples aged 21–65 in Canada [26]. However, no studies have yet used the ISS in CYC. The structure of the index was designed to use a unidimensional way; however, it revealed five possible distinct factors with the analyses of Mark et al. [21]. Although using ISS has some advantages, it is necessary to verify its reliability and validity before using it to study sexual satisfaction in this population. 

The current study is part of a project exploring sexual satisfaction among couples with at least one child aged 6 or younger. The study aims to construct a scale to measure sexual satisfaction in CYC by verifying the reliability and validity of a Japanese translation of the ISS in a CYC sample.

## 2. Materials and Methods

### 2.1. Design and Participants

This study used a cross-sectional design. For inclusion, participants needed to be (1) married Japanese heterosexual mothers or fathers living in Japan, (2) more than 20 years old (which constitutes adulthood in Japan), (3) parents of at least one child aged 6 or younger (pre-school age), and (4) biological parents of their child/ren. Young mothers or fathers under 20 years old, same-sex couples, and step-families were thus excluded from the study.

The participants were recruited during free health check-ups arranged by municipalities for 18-month-olds and 3-year-olds at community health centers. We contacted municipalities in several cities and towns of different population sizes to ensure diversity, requesting their cooperation through phone calls or visits. Five local municipalities of different locations and sizes (one capital city, two suburban cities, one suburban town, and one countryside city) agreed to participate. As none of these municipalities was in a metropolitan city, we approached other organizations involved with families with young children and secured permission from a non-profit organization to recruit participants at two family festivals in the metropolitan city of Tokyo.

### 2.2. Measure

The ISS was developed to evaluate the quality of sexual relationships in couples based on its authors’ [20] clinical and personal experience (Appendix A). Scores can be used as a measure of the degree of global sexual satisfaction or discord within a dyadic sexual relationship. The scale comprises 25 items, of which 12 are positive and 13 negative statements about various aspects of sexual life (e.g., “I feel that my partner enjoys our sex life”). Self-report responses were given on a 5-point Likert scale from 1, Rarely or never, to 5, Most or all of the time. For this study, all negatively worded items were reverse-scored, and the total score was computed. The higher the sum, the higher the sexual satisfaction.

#### Translation of the ISS into Japanese

The ISS [20] was translated into Japanese using the procedure recommended by the World Health Organization [27]. Translation was performed by a panel of four expert researchers: two Japanese authors of this study who are proficient in English (A, author 1 and B, author 5), and one English–Japanese bilingual American researcher (C) and one Hindi–English–Japanese trilingual Indian researcher (D) who were not otherwise involved in the project. Researchers A and C made the first Japanese version of the ISS using the forward- and back-translation technique with discussion between them. Researcher D checked the initial version, comparing it with the English ISS, and A and D made the second version after discussion. Researcher B then checked the second version, focusing on how natural the items sounded in Japanese, and then, A and B made the final Japanese version.

A pilot study using the final Japanese version of the ISS was conducted to check whether any of the items were ambiguous. Survey questionnaires of the ISS were given to 26 parents, mothers or fathers, who agreed to collaborate with this research at a free health checkup for their child (18–36 months [3 years] of age) with the cooperation of one municipality; 17 mothers and 17 fathers with at least one child aged 6 years or younger mailed back questionnaires. The responses indicated that no changes were required to the final Japanese version of the ISS.

### 2.3. Data Collection

The participants were recruited at 33 health check-ups in five community health centers and two family festivals between November 2017 and December 2018. Recruitment involved explaining the purpose and content of the survey orally and in writing to a parent (mother or father) who might consider participating and then giving them two handouts, one for them and one for their partner. Each handout contained a letter of invitation, a summary of the study project, information about ethical considerations, instructions for completing the questionnaire, a stamped return envelope, and the questionnaire. The questionnaire included questions regarding demographics, items from the ISS, and other variables related to sexual satisfaction (e.g., relationship satisfaction and parenting stress). Participation in this study was on an individual rather than a couple basis; each participant was instructed not to discuss the questionnaire with their partner and return the anonymously filled-out questionnaire separately via the postal service.

### 2.4. Factor Analyses of the Japanese Version of the ISS

Since items were worded both positively and negatively, the negatively worded items were reverse-scored before analysis. All the returned questionnaires were numbered and randomly divided into two groups by odd-even splitting. The data from odd-numbered questionnaires were used to perform an exploratory factor analysis (EFA) to determine the factors measured and their associated items. We then examined the factor structure and reliability of the resulting scale. The data from the even-numbered questionnaires were used to perform a confirmatory factor analysis (CFA) to evaluate model fit using the factor structure extracted by EFA, and the construct validity of the scale was examined. Factorial analyses were performed using SAS 9.4 software (SAS Institute Inc., Cary, NC, USA), in which the FACTOR procedure was used for EFA and the CALIS procedure for CFA.

#### 2.4.1. EFA and Reliability

Before factorial analyses, the data were checked for ceiling and floor effects based on the cut-off criterion used in Labarere et al. [28]. Factor analysis was then performed on the polychoric correlation matrix, as recommended where items are rated on a Likert-like scale [29,30]. The variance inflation factor (VIF) was used to check for multicollinearity [31,32]. As the data were ordinal Likert-type ratings, EFA was performed using the unweighted least squares (ULS) estimation method [33,34] with promax rotation [35]. The Kaiser-Meyer-Olkin (KMO) test of sampling adequacy, Kaiser’s rule, and Cattell’s scree test were applied to select which items to retain [36]. Factor loadings, cross-loadings, and communalities were confirmed based on recommended values.

The reliability of the model developed by EFA was assessed using polychoric ordinal alpha coefficients (which are considered better reliability estimates than alpha coefficients for Likert-type ratings) [37,38] and item-rest correlation coefficients (the association of an item with the total score on the other items) [39]. The discrimination of items in the scale was evaluated using good–poor analysis, in which a significant difference between two groups comprising the lowest 25% and highest 75% of scores confirm item discrimination [40].

#### 2.4.2. CFA and Construct Validity

The factorial validity of the scale structure indicated by EFA was assessed by performing CFA on the polychoric correlation matrix using the ULS estimation method [41]. Goodness of fit and construct validity were assessed. Model fitness was evaluated using component fit indices because the chi-square value is sensitive to sample size [42]. The construct validity of the proposed scale was assessed by investigating convergent and discriminant validity [43]. Composite reliability (CR; a measure of internal consistency) and average variance extracted (AVE; a summary indicator of convergence) were evaluated to test convergent validity [43]. The Fornell–Larker (F–L) criterion was used to assess discriminant validity [44].

## 3. Results

### 3.1. Participant Characteristics

Of 1,386 questionnaires handed out to parents, 593 were returned via mail (response rate: 42.8%). Of these, 588 were used for analysis; five were excluded because the respondents were single parents, were remarried with a child, or had left most questions unanswered. Participants ranged in age from 21 to 49 years old, with a mean of 34.6 (*SD* = 5.05) years; 53.7% (*n* = 316) were female, and 46.4% (*n* = 269) had entered college education. The odd- and even-numbered groups of participants created for factorial analysis each comprised 294 respondents. This was an adequate sample size for 25 questionnaire items based on the general rule that factor analysis should be performed using data from a minimum of five times as many participants as items (≥125 respondents in this case) [36]. As for the demographic variables of the two groups, there were no associations found by using Cramer’s V and Yule’s Phi, and no significant differences (Table 1).

### 3.2. Factor Analyses of the Japanese Version of the ISS

#### 3.2.1. EFA and Reliability

The results of the analyses were interpreted using the cut-off values quoted in parentheses. Floor effects (≥50% of scores being in the lowest response category) [28] were seen for six items (58.9% for item 4, 67.5% for item 5, 50.0% for items 13 and 14, 72.6% for item 15, and 52.9% for item 20), so these items were excluded. No ceiling effects were observed. No VIF value exceeded 5 [31,32], indicating no multicollinearity. Repeated EFAs were conducted using responses on the remaining 19 items from those in the odd-numbered group to identify the best factorial structure that satisfies all the criteria. Consequently, two factors comprising 11 items were extracted. The KMO value was 0.89 (>0.50), indicating that the sample had high factorability [36,45]. Two factors with eigenvalues of 5.7 and 1.5 (>1.0) [36,46] and accounting for 66.1% (≥60%) of the total variance [36] were identified based on Cattell’s scree test. The first factor was labeled “sexual satisfaction” and the second, “sexual dissatisfaction” (Table 2). All factor loadings were 0.60 or greater (sig. ≥ 0.50) [36], and no cross-loadings on the other factor exceeded 0.40 [47]. Every item had a satisfactory level of communality (≥0.40) [48].

The polychoric ordinal alpha coefficients were 0.89 for factor 1, 0.78 for factor 2, and 0.89 for the entire scale, indicating good internal consistency reliability (all ≥ 0.70) [37,49]. All item-rest correlation coefficients were between 0.33 and 0.78 (>0.30) [39], indicating that the scale had adequate internal consistency and reliability [50]. There was a significant difference between the two groups comprising the lowest 25% and highest 75% of scores for all items, indicating that they were discriminative [40].

#### 3.2.2. CFA and Construct Validity

**Figure 1:**
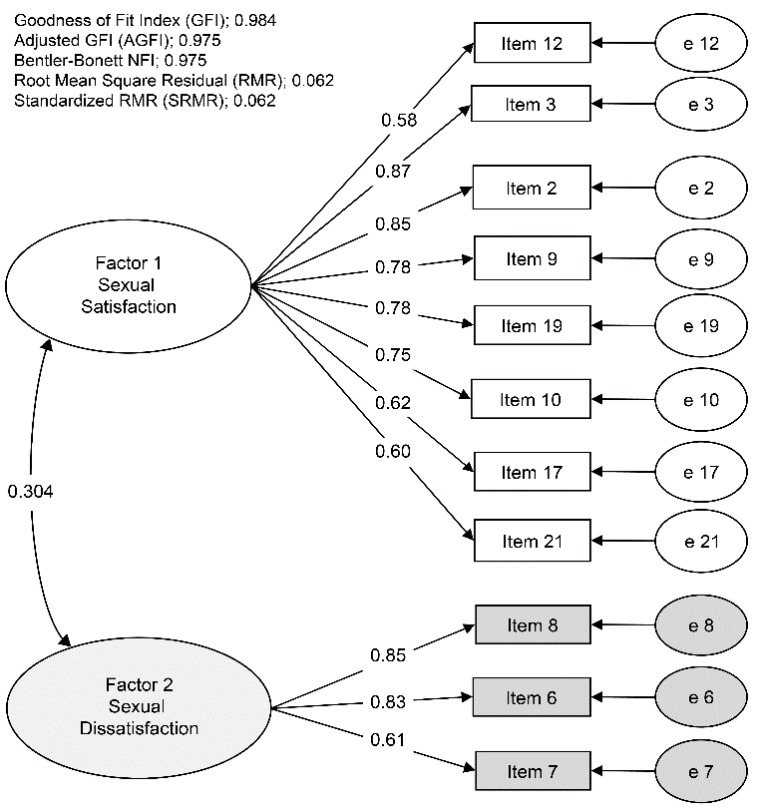
Confirmatory factor analysis of the revised Japanese version of the Index of Sexual Satisfaction. Data drawn from the even-numbered group (*N* = 294). Numbers superimposed on lines show the standardized loading of each item and the standardized covariance between factors 1 and 2; all *p* < 0.001.

The CFA of the revised scale model using the ULS estimation method was applied to a polychoric correlation matrix of the data of even-numbered participants. All the values supported the validity and fit of the model; the standardized loadings (all ≥ 0.50) [43]; the goodness of fit index, adjusted goodness of fit index, and Bentler–Bonett normed fit index (all good ≥ 0.90) [42]; and root mean square residual and standardized root mean square residual (both acceptable ≤ 0.08) [42] (Figure 1).

Construct validity was demonstrated by tests indicating satisfactory convergent and discriminant validity (Table 3). The CR (adequate ≥ 0.70) [32] and AVE (adequate ≥ 0.50) [43] values indicate adequate convergent validity. Discriminant validity was confirmed by the Fornell–Larcker (F–L) criterion: each AVE value was greater than the squared correlation between factors 1 and 2 [44].

## 4. Discussion

This study successfully developed and verified the Japanese version of the Index of Sexual Satisfaction for Couples with Young Children (ISS-CYC-J) through translation, factor analysis, and assessments of reliability and validity.

### 4.1. The Two-Factor Scale of the ISS-CYC-J

This two-factor scale measures sexual satisfaction with eight items and sexual dissatisfaction with three items. The two-factor structure of the ISS-CYC-J broadens the scope of examinations of couples’ sexual satisfaction, as it enables the relationship between sexual satisfaction and sexual dissatisfaction to be explored. The subtle feelings of ambivalence (being sexually satisfied and, at the same time, sexually dissatisfied) and indifference (not being sexually satisfied but also not sexually dissatisfied) can be evaluated [51]. The ISS-CYC-J could be utilized in research looking at longitudinal changes in the relationship between sexual satisfaction and dissatisfaction as well as the level of sexual satisfaction and dissatisfaction. Previous studies have noted that bi-dimensional scales of satisfaction and dissatisfaction give useful insights into sexual relationship quality in sexually active individuals with a mean age of 27.0 (*SD* = 9.4) [51] and relational quality [52,53,54].

The number of items was decreased from 25 items in the original ISS to 11 items in the ISS-CYC-J. Six items (4, 5, 13, 14, 15, and 20) were omitted prior to EFA due to a floor effect. More than half of participants (50.0%–72.6%) answered “none or rarely” for those items. Notably, those items described extreme states of sexual dissatisfaction, such as “I feel that sex is dirty and disgusting” (5), and “My partner is too rough or brutal when we have sex” (15). The ISS was created with reference to clinical experience of couples experiencing sexual problems [20]. We speculate based on the high percentages selecting the lowest value that many of our participants did not have such extreme sexual concerns.

Repeated EFAs indicated that eight items (1, 16, 22, 23, 11, 18, 24, and 25) did not load strongly onto any factor. They were thus excluded from the scale. These items related to the respondent’s partner’s happiness, such as “I feel that my partner enjoys our sex life” (1), and behavior, such as “My partner does not want sex when I do” (18). Thus, the ISS-CYC-J assesses “my” satisfaction and dissatisfaction with various aspects of sex life and should be used as a scale to measure self-focused evaluations. Interestingly, the New Sexual Satisfaction Scale contains two sub-scales: the ego-centered subscale measuring personal experiences, and the partner-/sexual activity-centered subscale measuring the partner’s behavior and the couple’s sexual activities [55]. The two dimensions of self-focused and partner-focused evaluations are distinct [55,56]. Thus, the bi-dimensional ISS-CYC-J constitutes the self-focused part of the original scale.

### 4.2. Strengths, Limitations, and Future Directions

The mean age and educational background of the study participants were approximately equivalent to those of CYC in the Japanese population. Their mean age was 34.6, and the mean age of Japanese mothers and fathers with at least one child aged 6 years or younger was 32.7 at the time of the survey [57]. The percentage enrolled in university was 46.4%. Considering the mean age of the participants and its standard deviation, their years of entering university were 2001 through 2011. The mean percentage of university enrollments in those years in the Japanese population was 45.7% [58]. Thus, this sample could be considered representative of CYC in the general Japanese population.

Few studies have yet examined sexual satisfaction in CYC. Moreover, to the best of our knowledge, no valid Japanese tool has been created for assessing sexual relationships, including those of CYC. Hence, the ISS-CYC-J has the potential to advance our understanding of CYC sexual relationships. The characteristics of the current study, however, do limit the applicability of the scale. The scale has been validated in Japanese heterosexual CYC couples with a mean age of 34.6. To detect if the revised Japanese version of the ISS is the same structure among mothers and fathers, the next step of our study is necessary to assess the equivalence of the scale across mothers and fathers using multi-sample confirmatory factor analysis. Additionally, further studies are needed to evaluate its validity in step-families, for parents under 20 years old, and in same-sex couples. As there is a trend toward delayed childbearing [59], it is also important to conduct future studies in older CYC, since they might have different sexual issues than younger couples. To enable studies of sexual satisfaction in CYC in other socio-cultural contexts than Japan, an English version of the CYC-ISS will also need to be verified due to the impact of culture on sexuality [60].

## 5. Conclusions

The present study developed the Japanese version of the Index of Sexual Satisfaction for Couples with Young Children (ISS-CYC-J) and demonstrated its reliability and validity. The scale will help future studies investigate sexual satisfaction in couples raising young children and explore its associations with other variables, such as relationship satisfaction and well-being.

## Figures and Tables

**Table 1:** Demographic Characteristics *(N* = 588).

Baseline Characteristic	Item	Odd-Numbered Group	Even-Numbered Group	Effect Size	*p*-Value
		*n* = 294	*n* = 294		
		*n* (%)	*n* (%)		
Participants	Mother	158 (53.7)	158 (53.7)	0 ^1^	1.000 ^4^
	Father	136 (46.3)	136 (46.3)		
Age (years)	(Mean ± SD)	(34.5 ± 5.06)	(34.7 ± 5.05)	0.023 ^2^	0.545 ^5^
Period of marriage (years)	(Mean ± SD)	(6.17 ± 3.03)	(6.34 ± 3.29)	0.020 ^2^	0.626 ^5^
Job	None	68 (23.5)	68 (23.4)	0.035 ^3^	0.860 ^4^
	Full time (regular)	177 (61.2)	185 (63.6)		
	Full time (nonregular)	8 (2.8)	8 (2.7)		
	Part time	36 (12.5)	30 (10.3)		
Education	Junior high/senior high school	60 (20.8)	66 (22.7)	0.020 ^2^	0.638 ^5^
	Technical school/junior college	93 (32.2)	92 (31.6)		
	Four-year college/graduate	136 (47.0)	133 (45.7)		
Economic conditions	Very good	9 (3.1)	10 (3.4)	0.015 ^2^	0.713 ^5^
	Good	126 (43.3)	114 (39.3)		
	Not very good	132 (45.4)	150 (51.7)		
	Bad	24 (8.2)	16 (5.6)		
Number of child(ren)	One	105 (35.7)	95 (32.4)	0.049 ^2^	0.238 ^5^
	Two	139 (47.3)	138 (47.1)		
	Three	45 (15.3)	50(17.1)		
	Four or more	5 (1.7)	10 (3.4)		

Notes; SD: standard deviation. The total number for each characteristic is different owing to missing values. ^1^ Yule’s Phi. ^2^ Glass’s r. ^3^ Cramer’s V. ^4^ Chi-square test. ^5^ Wilcoxon rank-sum test.

**Table 2:** Exploratory factor analysis and reliability estimates of the revised Japanese version of the ISS *(N* = 294 ^1^).

Item Number	Item	EFA			IRC ^2^	Good-Poor Analysis ^3^			
		Factor 1	Factor 2	C		<25% *n =* 69		>75% *n* = 81	
		**FL**	**FL**			Mean	*SD*	Mean	*SD*
**Factor 1: Sexual Satisfaction**									
12	I think that sex is wonderful.	0.82	−0.33	0.50	0.47	2.63	1.01	4.16	0.70
3	Sex is fun for my partner and me.	0.78	0.15	0.76	0.78	2.04	0.70	4.16	0.66
2	My sex life is very exciting.	0.76	0.13	0.70	0.74	1.73	0.67	3.97	0.75
9	My partner is sexually very exciting.	0.74	0.12	0.65	0.71	1.95	0.76	3.94	0.73
19	I feel that our sex life really adds a lot to our relationship.	0.72	0.03	0.54	0.64	2.46	0.98	4.46	0.61
10	I enjoy the sex techniques that my partner likes or uses.	0.70	0.13	0.60	0.68	1.86	0.74	3.71	0.91
17	I feel that sex is a normal function of our relationship.	0.67	0.03	0.47	0.60	2.29	1.05	4.48	0.61
21	It is easy for me to get sexually excited by my partner.	0.60	0.10	0.43	0.57	1.77	0.74	3.58	1.01
**Factor 2: Sexual Dissatisfaction**									
8	I feel that my sex life is lacking in quality.	0.04	0.83	0.72	0.53	2.66	1.15	4.29	0.75
6	My sex life is monotonous.	0.06	0.80	0.69	0.54	2.26	1.05	3.91	0.87
7	When we have sex it is too rushed and hurriedly completed.	−0.09	0.68	0.40	0.33	2.86	1.13	4.04	0.86

Notes; ISS: Index of Sexual Satisfaction, EFA: exploratory factor analysis, FL: factor loadings, C: communality, ^1^ Odd-numbered group. ^2^ IRC: item-rest correlation was conducted using Spearman’s correlation coefficient; all *p* < 0.001. ^3^ Good–poor analysis was conducted using the Wilcoxon rank-sum test; all *p* < 0.001.

**Table 3:** Construct validity of the revised Japanese version of the ISS (*N* = 294 ^1^).

Factor	CR	AVE	F–L Criterion	
			F1	F2
F1	0.903	0.557	***0.557***	
F2	0.814	0.598	0.228	***0.598***
Scale	0.932	0.558		

Notes; ^1^ Even-numbered group, ISS: Index of Sexual Satisfaction, CR: composite reliability, AVE: average variance extracted, F–L criterion: Fornell–Larcker criterion; each AVE estimate is presented diagonally in bold italic type and the off-diagonal number is the squared correlation between factors (Spearman’s correlation coefficient; *p* < 0.001).

## Data Availability

Not applicable.

## The use of the ISS scale

Permission has been obtained from the Walmyr Publishing Company to use the original scale of the Index of Sexual Satisfaction for this study project.

## Appendix A

**Table A1:** Index of Sexual Satisfaction (Hudson et al., 1981).

**	1	I feel that my partner enjoys our sex life
	2	My sex life is very exciting
	3	Sex is fun for my partner and me
*	4	I feel that my partner sees little in me except for the sex I can give
*	5	I feel that sex is dirty and disgusting
	6	My sex life is monotonous
	7	When we have sex it is too rushed and hurriedly completed
	8	I feel that my sex life is lacking in quality
	9	My partner is sexually very exciting
	10	I enjoy the sex techniques that my partner likes or uses
**	11	I feel that my partner wants too much sex from me
	12	I think that sex is wonderful
*	13	My partner dwells on sex too much
*	14	I feel that sex is something that has to be endured in our relationship
*	15	My partner is too rough or brutal when we have sex
**	16	My partner observes good personal hygiene
	17	I feel that sex is a normal function of our relationship
**	18	My partner does not want sex when I do
	19	I feel that our sex life really adds a lot to our relationship
*	20	I would like to have sexual contact with someone other than my partner
	21	It is easy for me to get sexually excited by my partner
**	22	I feel that my partner is sexually pleased with me
**	23	My partner is very sensitive to my sexual needs and desires
**	24	I feel that I should have sex more often
**	25	I feel that my sex life is boring

Response options: 1: Rarely or none of the time, 2: A little of the time, 3: Some of the time, 4: Good part of the time, and 5: Most or all of the time. Notes; Items 4, 5, 6, 7, 8, 11, 13, 14, 15, 18, 20, 24, and 25 are negatively worded. * Items removed owing to a floor effect. ** Items removed based on exploratory factor analysis.
